# 808 Combination of Adipose Micro Fragments and Liquid Scaffold Improve Wound Healing Outcomes

**DOI:** 10.1093/jbcr/irac012.356

**Published:** 2022-03-23

**Authors:** Sara Sheikh-Oleslami, Ida Hassanpour, Nafise Amiri, Reza B Jalili, Ruhangiz Taghi Kilani, Aziz Ghahary

**Affiliations:** The University of British Columbia, North Vancouver, British Columbia; Burn and Wound Healing Research Lab, The University of British Columbia, Vancouver, British Columbia; University of British Columbia, Burn and Wound Research Group, Vancouver, British Columbia; University of British Columbia, Vancouver, British Columbia; UBC, Vancouver, British Columbia; UBC, Vancouver, British Columbia

## Abstract

**Introduction:**

In full-thickness wounds, high levels of inflammation, lack of matrix deposition and paucity of progenitor cells delays normal healing processes. One major problem with commercially available solid (sheet) scaffolds is their inability to conform to wounds of varying shapes and sizes. To overcome this, we previously generated a liquid, injectable skin substitute which can fill wounds of any shape and depth from bottom up and has all the necessary ingredients for skin cells to be nourished, proliferate, and migrate in. In combination with adipose micro fragments as a viable source of progenitor cells, a composite, *in situ *forming skin substitute was tested for treatment of silicon ring splinted wounds in rats.

**Methods:**

The *in vitro* survival and migratory capacity of adipocytes derived from rat micro-fragmented fat when cultured in our 3D nutritional scaffold was examined with a Live/Dead assay. The efficacy of our combined liquid scaffold alone (MF) or with adipose micro fragments (MFA) in treating full thickness splinted wounds in rats was compared to a standard dressing protocol (NT). The healing process was monitored for 10 days. Following wound measurements, histological and immunofluorescent analyses were performed and compared.

**Results:**

Adipose-derived cells migrated within the 3D nutritional liquid scaffold after 7 and 14 days. The number of red (dead) cells was negligent, indicating cell viability. *In vivo*, both MFA and MF showed both accelerated and ameliorated wound healing, including complete epithelialization and less immune cell infiltration, compared to the NT control. No significant differences were observed between the MF and MFA groups for any outcome.

**Conclusions:**

Our findings show that a 3D nutritional liquid skin scaffold is a rich environment for adipocyte viability and migration and that addition of adipose micro fragments to this scaffold can be used as a rich source of cells.

